# A Multi-Scale Approach to Membrane Remodeling Processes

**DOI:** 10.3389/fmolb.2019.00059

**Published:** 2019-07-23

**Authors:** Weria Pezeshkian, Melanie König, Siewert J. Marrink, John H. Ipsen

**Affiliations:** ^1^Groningen Biomolecular Sciences and Biotechnology Institute and Zernike Institute for Advanced Materials, University of Groningen, Groningen, Netherlands; ^2^Department of Physics, Chemistry and Pharmacy, Center for Biomembrane Physics (MEMPHYS), University of Southern Denmark, Odense, Denmark

**Keywords:** dynamic triangulated surfaces, Martini coarse-grain simulation, Shiga toxin, simulation of continuum model, membrane remodeling, implicit protein model

## Abstract

We present a multi-scale simulation procedure to describe membrane-related biological processes that span over a wide range of length scales. At macroscopic length-scale, a membrane is described as a flexible thin film modeled by a dynamic triangulated surface with its spatial conformations governed by an elastic energy containing only a few model parameters. An implicit protein model allows us to include complex effects of membrane-protein interactions in the macroscopic description. The gist of this multi-scale approach is a scheme to calibrate the implicit protein model using finer scale simulation techniques e.g., all atom and coarse grain molecular dynamics. We previously used this approach and properly described the formation of membrane tubular invaginations upon binding of B-subunit of Shiga toxin. Here, we provide a perspective of our multi-scale approach, summarizing its main features and sketching possible routes for future development.

## Introduction

Many biological processes involve large scale changes in lateral chemical organization and geometrical shapes of biological membranes (McMahon and Boucrot, 2015; Chavent et al., 2018). The modeling of these processes, by computer simulation, is a challenging task since they typically involve a wide range of length and time scales that cannot be captured in full by any single current simulation technique (Enkavi et al., 2019; Marrink et al., 2019). At large length scales, computational, and analytical techniques based on continuum models have played a great role in our understanding of these processes and has revealed many important generic phenomena (Seifert et al., 1991; Bozic et al., 1992; Ramakrishnan et al., 2013, 2015). Nevertheless, these predictions are often obscured by the simplicity of the model and by the approximations needed to make them mathematically tractable. In addition, such phenomenological models contain few model parameters that are typically hard to relate to their molecular origin. At small length scales, particle-based computer simulations techniques e.g., molecular dynamics (MD) and dissipative particle dynamics (DPD), are robust techniques to elucidate complex membrane behaviors but with a limited capacity to predict large length scale cooperative phenomena (Gao et al., 2007; Li et al., 2016; Enkavi et al., 2019; Marrink et al., 2019). To overcome these limitations, we have used a multi-scale simulation procedure that bridges the gap between the particle and continuum based models and allows the simulation of large biological membrane patches while retaining details from the atomistic length scale (Pezeshkian et al., 2016). Here, we summarize the main features of the method, extend its capacity to describe a wider range of processes and sketch possible routes for further development.

## Methods

In our multi-scale approach, the large-scale physical properties of a membrane are described by a coarse-grained model which captures the elastic energy of membrane conformations and the energetics of the lateral organization of its chemical constituents. Such a model only contains a few model parameters which are calibrated using atomistic and mesoscopic simulations (Marrink et al., 2007).

### Simulation of Continuum Model

**Figure 1:**
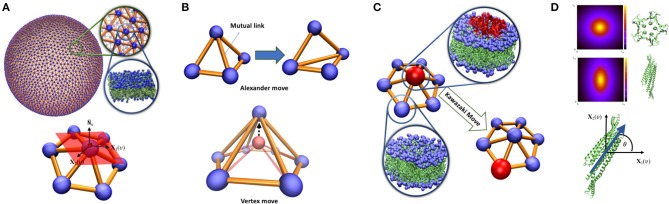
**(A)** Triangulated surface representation of a vesicle. Each vertex represents a segment of a bilayer containing hundreds of lipids. On each vertex, surface normal ($\hat{\text{N}}$), principal directions **X**_1_(υ), **X**_2_(υ) and their associated principal curvature (*c*_1υ_, *c*_2υ_) can be determined. **(B)** Top: Alexander move, mutual link between neighboring triangles is flipped and two new triangles are generated. Bottom: vertex move, a chosen vertex (red bead) is moved in a random direction **(C)** Proteins are modeled as an inclusion that can lay on a vertex and can jump to the neighboring vertex via Kawazaki moves. **(D)** Vertex based model of curvature map induced by proteins with lateral symmetry higher than 2 (top) and π-symmetric proteins (middle). The inclusion associated with these proteins should have a character of a two-dimensional vector in the plane of a vertex (bottom). The angle between the protein direction and the membrane main principal direction.

A continuous membrane is discretized by a dynamical triangulated surface (DTS) containing *N*_υ_ vertices, *N*_*T*_ triangles, *N*_*L*_ links which together form an irregular planer triangulated network (Figure 1A). The difference between dynamical and static triangulation is that the mutual link between two neighboring triangles can flip (Alexander moves). This allows to sample through all possible triangulations for a given *N*_υ_, *N*_*T*_, *N*_*L*_. Link flipping and positional updates of the vertices gives the fluid character with full translational invariance in the plane of the surface (Figure 1B). In this representation, a vertex can be visualized as a segment of a bilayer containing hundreds of lipids, this means that the resolution of the model is limited to the length-scales above few nanometers. To ensure self-avoidance of the surface each vertex is equipped with a spherical bead. Using a set of discretized geometrical operations, each vertex is furthermore assigned with a normal vector ${\hat{\text{N}}}_{\upsilon }$, surface area *A*_υ_ (one third of the area of its neighboring triangles), principal curvatures (*c*_1υ_, *c*_2υ_) and principal directions (**X**_1_(υ), **X**_2_(υ)) (Ramakrishnan et al., 2010) (Figure 1A). This suffices to construct an elastic energy function associated with membrane bending that allows us to obtain the surface equilibrium configurations using numerical update algorithms. In this work, we have employed the Metropolis Monte Carlo algorithm (Ramakrishnan et al., 2010; Bahrami et al., 2012; van der Wel et al., 2016), but many other updating schemes are possible (Noguchi and Takasu, 2001; Cooke et al., 2005; Noguchi and Gompper, 2006; Peng et al., 2013; Mauer et al., 2018).

### Elastic Energy

The Helfrich Hamiltonian (Helfrich, 1973) is the classic approach to describe membrane shape phenomena. The membrane elastic energy *E*_*b*_ can be expressed in the terms of two surface invariants, the mean curvature *H* = 0.5(*c*_1_ + *c*_2_), and Gaussian curvature, *K* = *c*_1_*c*_2_. A discretized form of the Helfrich Hamiltonian can be written as:

(1) $$\begin{array}{l} E_{b}=\frac{\kappa }{2}\overset{N_{\upsilon }}{\underset{1}{\sum }}{\left(2H_{\upsilon }-\;{\bar{C}}_{0}\right)}^{2}A_{\upsilon }+\kappa_{G}\overset{N_{\upsilon }}{\underset{1}{\sum }}K_{\upsilon }A_{\upsilon }\; \end{array}$$

The second term of this equation only depends on the surface topology and does not change by continuous membrane deformation (Gauss-Bonnet theorem). The mean curvature elastic constant κ is called the bending elasticity, which carries the dimension of energy. The constant ${\bar{C}}_{0}$ is called the spontaneous curvature, which represents a possible asymmetry between the two monolayers, e.g., differing solvent conditions. ${\bar{C}}_{0}=0$ for a symmetric membrane. Equation (1) can be expanded in numerous ways depending on the membrane process at play. For example, for processes where a significant part of the total membrane surface undergoes deformations much faster than the flip flop rate of any monolayers chemical component, a monolayer area difference elastic term must be included (Seifert et al., 1991; Bozic et al., 1992). The difference in the area of the monolayers can be obtained as

(2) $$\begin{array}{l} \Delta A=h\overset{N_{\upsilon }}{\underset{\upsilon }{\sum }}2H_{\upsilon }A_{\upsilon } \end{array}$$

Where *h* is the membrane thickness. Up to second order, the area-difference elastic energy is expressed as $E_{s}={\frac{k_{r}}{2{h^{2}A}_{0}}\left(\Delta A-{\Delta A}_{0}\right)}^{2}$, with *k*_*r*_ denoting the area compression modulus (Svetina and Žekš, 2014). Another relevant energy term that can be included is the elastic energy associated with change in the volume (*V*) of a closed surface (vesicle), $E_{V}=\frac{K_{V}}{2V_{0}}{\left(V-V_{0}\right)}^{2}$ where both the volume compression modulus *K*_*V*_ and the equilibrium volume *V*_0_ are set by the osmotic conditions of the solvents in an experiment. For a triangulated surface, the volume can be easily obtained as

(3) $$\begin{array}{l} V=\frac{1}{3}\overset{N_{T}}{\underset{T=1}{\sum }}\left({\vec{\text{R}}}_{T}.{\hat{\text{N}}}_{T}\right)A_{T} \end{array}$$

Here, ${\vec{\text{R}}}_{T}$ is the position of any point on the triangle *T*, ${\hat{\text{N}}}_{T}$ and *A*_*T*_ are the normal vector and area of the oriented triangle *T*, respectively. For analysis of bounded membrane patches or semi-flat membranes in a periodic boundary box, a contribution τ*A*_*p*_ to the energy in Equation (1) becomes important. *A*_*p*_ and τ are the projected area and frame tension of the membrane, respectively.

When we are dealing with membranes with highly curved regions, e.g., formation of narrow necks prior to scission during a fission process, Equation (1) requires modification. In these regions, the curvatures of different monolayers can be significantly different. A practical approach to include this mismatch is to treat the bending energy associated with each monolayer separately. Using mid-plane principal curvatures, the mean curvature of each of the monolayers can be determined as Safran (1994):

(4) $$\begin{array}{l} H_{up}=\frac{H+2Kh}{1+hH+Kh^{2}},\;H_{low}=\frac{-H+2Kh}{1-hH+Kh^{2}} \end{array}$$

### Implicit Protein Model

Membrane proteins can locally influence bilayer shape through direct and indirect couplings. Direct impacts include local rigidification (Zhang et al., 2015), local membrane curvature imprint (Pezeshkian et al., 2017b; Corradi et al., 2018; Wang et al., 2018), local change in membrane thickness (Corradi et al., 2018) etc. Indirect effects arise from their interactions with other proteins that have the capacity to affect the membrane shape through cooperative phenomena. In our multi-scale simulation approach, these couplings are identified and quantified through atomistic and mesoscopic simulations and they are included in the system energy as new terms added to Equation (1). In the modeling, a protein or nanoparticle (an inclusion) is assigned to a vertex in the triangulation. Each vertex can at most occupy one inclusion, which naturally handles the in-plane excluded volume effect between inclusions. It also introduces a natural length scale to the model since we can associate the smallest possible area of a vertex with the projected area of the inclusion in question. Inclusions can move laterally through updates of the triangulation or by jumps between the neighboring vertices via Kawazaki moves (Figure 1C).

When an inclusion is situated in a vertex, it may change the elastic energy contribution from the vertex. For membrane proteins, the simplest model is to locally increase membrane bending rigidity (Frolov and Zimmerberg, 2008; Schweitzer et al., 2015). The most important effect of membrane proteins, that greatly influences the large-scale membrane shape, is to induce a local membrane curvature (Kozlov et al., 2014). This induced curvature can be in-plane rotationally symmetric or asymmetric. As a consequence of Eulers curvature formula, vertex-based inclusions, except π-symmetric inclusions (symmetric upon rotation by 180 degrees in the plane of the membrane, Figure 1D), can only induce symmetric curvature (Peliti and Prost, 1989). It may seem that this is a shortcoming of the model. Nevertheless, highly asymmetric curvature imprints decays quickly in the membrane plane (Dasgupta et al., 2017; Corradi et al., 2018) and does not appear in a macroscopic membrane model. The impact of these inclusions can be modeled by adding a local energy contribution *e*_υ_ = −κ*HC*_0_*A*_υ_ to the bending energy per vertex, where *C*_0_ is the local curvature imprint of the protein and needs calibration from finer scale simulations. Notice that *C*_0_ can only be identified with ${\bar{C}}_{0}$ in Equation (1) for a fully covered membrane. π-symmetric inclusions can locally bend the membrane differently in different directions (Frolov and Zimmerberg, 2008). Such inclusions can thus be given an orientation in the plane in the direction with maximal directional curvature imprint $\left(C_{0}^{∥}\right)$ while the perpendicular direction in the plane gives the lowest directional curvature imprint $\left(C_{0}^{⊥}\right)$ (Figure 1D). The membrane curvature in these directions can easily be obtained by Eulers curvature formula $C^{∥}=c_{1\upsilon }\cos^{2}\left(\theta \right)+c_{2\upsilon }\sin^{2}\left(\theta \right)$ and $C^{⊥}=c_{1\upsilon }\sin^{2}\left(\theta \right)+c_{2\upsilon }\cos^{2}\left(\theta \right)$ where θ is the angle between the orientation of the inclusion and the direction of the main principal curvature of the membrane. Such inclusion will give rise to an additional local contribution to the total elastic energy in Equation (1), $e_{\upsilon }=\left[\frac{\kappa_{1}}{2}{\left(C^{⊥}{-C}_{0}^{⊥}\right)}^{2}+\frac{\kappa_{2}}{2}{\left(C^{∥}-C_{0}^{∥}\right)}^{2}\right]A_{\upsilon }$, where κ_1_ and κ_2_ are the directional bending rigidities imposed by the inclusion on the membrane. To complete the modeling, we need to include interactions between the inclusions. Here, we will only focus on the pair interactions but nevertheless the method can be extended to multi-body interactions. The pair-interactions between inclusions can be divided into two types: (i) as a function of distance between the proteins in the 3-dimensional space, e.g., electrostatic and van der Waals forces, (ii) as a function of a distance alongside the geodesic direction between two inclusions in the membrane, e.g., membrane mediated interactions (Haselwandter and Wingreen, 2014; Johannes et al., 2018). The former type of interactions can be modeled simply by a constant interaction energy when two inclusions are in proximity in the 3D space. This is a practical and valid choice, since the resolution of the model is well-below a range to capture the protein specific interactions. The second type of interactions is more challenging since it depends on the local curvature of the membrane. A particular consequence of this is that interactions between two neighboring non-isotropic inclusions can first be calculated after parallel transport between them, where the in-plane orientations of the inclusion is kept fixed along their mutual geodesic curve (Ramakrishnan et al., 2010). The interaction between two inclusions on the neighboring vertices is only a function of angle between their in-plane orientations alongside geodesic direction: $\Delta \Theta =\Theta_{i}-{\Theta^{\prime }}_{j},\;where\;\Theta_{i}$ is the orientation of inclusion residing on vertex *i*, ${\Theta^{\prime }}_{j}\;$represents the orientation of inclusion residing on vertex *j* after parallel transport to vertex *i*. This energy function can be written in term of Fourier series as

(5) $$\begin{array}{l} \varepsilon_{ij}\left(\Delta \Theta \right)=-\varepsilon_{0}-\mu_{0}\overset{M}{\underset{k=1}{\sum }}\frac{a_{k}}{M}\cos\left[kQ_{ij}\Delta \Theta +\Xi_{k}\right]\; \end{array}$$

The first term (−ε_0_) models the isotropic part of the interaction between two inclusions while the second term is to model anisotropic interactions e.g., caused by steric factors and the distribution of the peptide groups in a protein (Domanski et al., 2017). *M* is a constant integer and its value depends on the chosen degree of coarse graining. Larger *M* allows to include more structural details of the protein shape in the interactions with other proteins. Ξ_*k*_ are the phase shift and _μ_0_*ak*_/*M* are amplitude of the Fourier modes and both need fitting from finer simulation techniques. By setting $\overset{M}{\underset{k=1}{\sum }}a_{k}=M$, μ_0_ can be defined as the lowest energy level of the anisotropic part of the interaction. *Q*_*ij*_ is the least common multiple of the degree of the *i,j* proteins symmetry in the plane of the membrane (*N*). Note that the interaction energy in Equation (5) can also be used to model lipid domain formations in multicomponent membranes (Ramakrishnan et al., 2010; Hansen et al., 2017).

Different approaches can be used to model proteins on triangulated surfaces e.g., introducing a curvature field and additional length scale to the model (Tourdot et al., 2014), however we prefer our procedure since it allows the calibration of all parameters solely through a bottom up approach. This increases the predication power of the model without need to tune the inputs parameters to reach the excepted outcome.

### Calibration

To start a DTS simulation for a membrane containing different lipids and proteins, all the mentioned model parameters need to be calibrated using results from experiments or simulations of finer scales. Below we discuss several of these parameters $\left(\kappa ,C_{0}^{∥},\;C_{0}^{⊥},\;\varepsilon_{0},\;\mu_{0},\;N,\;a_{k},\;\Xi_{k}\right)$.

*Bending rigidity* κ: Bending rigidity is known for many one component lipid bilayers from both experiment and simulations. However, for new lipid bilayers, fluctuation spectrum analysis is a powerful technique to extract this parameter. Both, coarse grained and all-atom MD simulation can be used to calibrate this parameter (Brandt et al., 2011; Watson et al., 2012; Venable et al., 2015).

*Local curvature imprint* $\left(C_{0}^{∥},\;C_{0}^{⊥}\right)$: All-atom MD simulation has proven successful for calibration of these model parameters (Pezeshkian et al., 2016, 2017b; Kociurzynski et al., 2019). From an MD simulation trajectory, membrane curvature can be measured using different approaches. An accurate method is to use the first moment of the lateral membrane pressure profile, κ*C*_0_ = ∫*z*Π(*z*)*dz* (Safran, 1994). However, this approach has several problems. First, a converged lateral pressure profile requires very long simulations even for pure membrane systems. Secondly, it only provides the mean value of the induced curvature ($C_{0}^{∥}+C_{0}^{⊥}$) unless the protein orientation is restricted (Bruhn et al., 2016; Ali Doosti et al., 2017). The second method is a geometrical approach and consists of fitting the upper and lower monolayer of the membrane to an analytical function and calculating the time-average curvature map on the surface of the bilayer. Note, since the typical radius of the curvature induced by proteins is much larger than a feasible MD simulation box size, the total average curvature of the fitted surface is zero. Therefore, one should only average the curvature of the surface up to a distance, in which the presence of the protein changes the lipid density, from the center of the proteins (Pezeshkian et al., 2016, 2017b; Corradi et al., 2018).

*Protein-protein interaction parameters* (ε_0_, μ_0_, *N, a*_*k*_, Ξ_*k*_): An efficient approach to calibrate these parameters is to use coarse grained MD or DPD simulations. Typically, large simulation boxes are needed because the system size should be large enough so that the proteins do not interact (including membrane-mediated interactions) with their periodic image. Secondly a long simulation is required to disentangle the diffusive approach from the systematic interaction. In addition, mesoscale simulations allow us to derive a potential of mean force (PMF) profile that can be used to calibrate (ε_0_, μ_0_) (de Meyer et al., 2008; Periole et al., 2012; Domanski et al., 2017). In-plane symmetry of the protein structure (*N)* can be found from the crystal structure. *a*_*k*_ and Ξ_*k*_ can be calibrated from both the density map or from free energy profile as a function of angle between the proteins.

### Example: Shiga Toxin Induced Tubular Membrane Invaginations

The bacterial Shiga toxin is a member of the AB_5_ protein family that is composed of an enzymatically active A-subunit, and a receptor-binding B-subunit. STxB is homopentameric and mediates intracellular toxin trafficking via binding to the glycolipid globotriaosylceramide (Gb3) at the plasma membrane of target cells. Shiga toxin can enter the cell by both clathrin-dependent and independent endocytosis. The formation of tubular membrane invaginations is the first step in the clathrin-independent STxB uptake (Römer et al., 2007). Previously, we have used this multi-scale simulation approach to describe formation of membrane tubular invaginations upon STxB binding. Here we shortly discuss the scheme and results.

**Figure 2:**
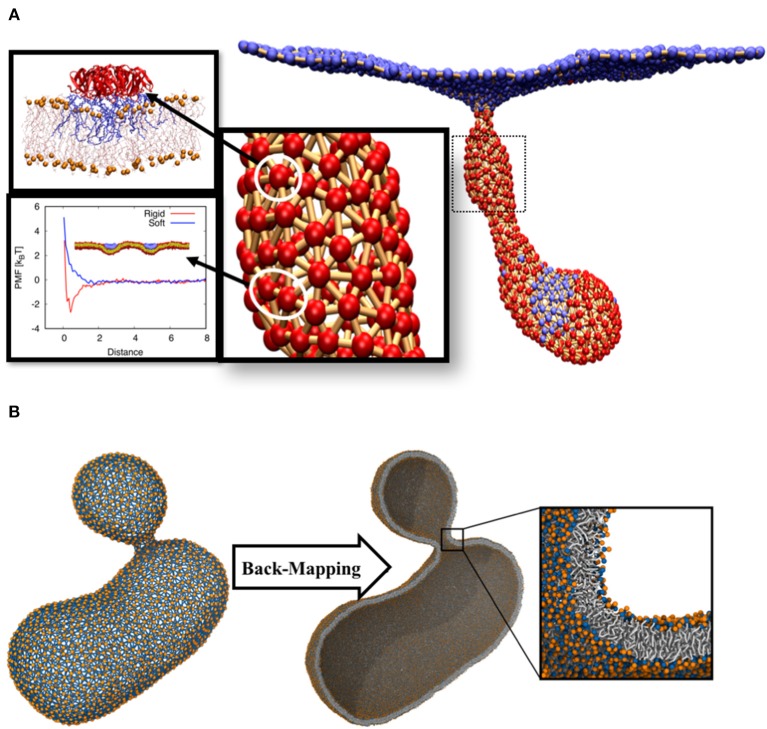
**(A)** Tubular membrane invaginations induced by Shiga toxin. The color code for the beads is the same as in Figure 1B. All atom molecular dynamic simulations were used to obtain local curvature induced by the protein (Pezeshkian et al., 2016). Protein-protein interaction was calibrated using DPD simulations. **(B)** The final structure of a budding vesicle after a DTS simulation is back-mapped to the particle based CG Martini model.

- Using the STxB crystal structure, we measured the projected area of STxB to be *38.5 nm*^2^ (we approximated the lateral shape as a circle). The smallest area of a vertex is equal to $\frac{\sqrt{3}}{2}l^{2}$ (*l* is the minimum length of a link, or a vertex size). Therefore, *l* ≈ 6.7 *nm*.
- Local curvature induced by STxB was measured using all atom MD simulations (Pezeshkian et al., 2016). The radius of curvature is found to be *R* ≈ 29.4 *nm* = 4.39 *l*.
- DPD simulation was used to find STxB-STxB interactions. Using this method and experiments on unilamellar vesicles, we provide evidence that thermal Casimir-like force arising from membrane surface fluctuations are responsible for STxB clustering (Pezeshkian et al., 2017a). The computed PMF profile shows that the potential depth, taken to represent the isotropic strength of the pair interaction, is around *2.5 k*_*B*_*T* (Figure 2A). STxB is a pentamer (2π/5-symmetric), therefore *N* = *5*. Based on these numbers, we defined the simplest form of the interaction energy as $\varepsilon_{ij}=-2.5+\left(1+\cos5\left[\Theta_{i}-{\Theta^{\prime }}_{j}\right]\right)$ in units of *k*_*B*_*T*.
- Using the above input parameters, we performed a Monte Carlo simulation of DTS in the constant frame tension ensemble (τ = 0) and could reproduce the behavior as seen in the experimental setups, namely formation of a tubular membrane invagination (Römer et al., 2007). We also found the minimum requirements for the formation of tubular membrane invaginations, i.e., (1) capacity of the individual proteins to induce local membrane curvature (2) their ability to cluster, by any mean, upon binding to the membrane (Figure 2A) (Pezeshkian et al., 2016).

## Back-Mapping to CG Model

The main assumption of this multi-scale simulation approach is that local properties of the membrane do not strongly get affected by large-scale membrane configurational changes. However, local lateral organizations of complex membranes chemical constituents can change upon large scale membrane deformations (Baoukina et al., 2018). In order to overcome this limitation, we have developed an algorithm that back-maps a DTS structure to its corresponding Martini CG model (Marrink et al., 2007; Marrink and Tieleman, 2013). This algorithm makes it possible to use DTS to equilibrate the slow large-scale membrane conformational change and exploit the Martini model to equilibrate the local lipid distributions. As a first attempt to explore this procedure, we performed a DTS simulation on a vesicle with a smaller volume/surface ratio of a perfect sphere (0.7) and a spontaneous curvature of 0.025 nm^−1^. Under this condition, the DTS simulation predicted the formation of a vesicular bud (Figure 2B) (Seifert et al., 1991; Markvoort et al., 2009; Bahrami et al., 2017). We then back-mapped the DTS structure to its corresponding Martini model and after a short energy minimization, it was ready for an MD simulation. The detail of this procedure is out of the scope of this article and will be published elsewhere.

## Summary and Perspectives

We described an extended version of our multi-scale simulation procedure that uses a bottom up scheme to calibrate DTS model parameters (Pezeshkian et al., 2016). The approach is well-suited for investigating membrane involved biological processes that take place at a large-range of time and length scales that cannot be captured by any single current simulation techniques.

One of the clear advantages of exploiting DTS at macroscopic length scales is the speed. DTS allows us to simulate micron size vesicles, decorated with membrane proteins, on a single CPU core. This length-scale is hardly reachable (using much more computational power) by any particle-based computer simulation techniques (Cooke et al., 2005; Ayton and Voth, 2009). Nevertheless, the approach still suffers from several limitations that need to be resolved. For example, DTS simulations with dynamic topology has been only developed for several special purposes (Jeppesen and Ipsen, 1993; Shillcock and Boal, 1996; Gompper and Kroll, 1998; Shillcock and Seifert, 1998) that limits its applications, as a generic method, to describe processes that involve membrane topological changes, e.g., membrane scission and poration (Boye et al., 2017). Another limitation is the current implicit protein model that is only applicable for membrane proteins. One possibility is to adopt one protein to few beads strategy e.g., essential dynamics coarse-graining (Zhang et al., 2008) to extend the range of the DTS protein mapping. Another route to increase the molecular level detail is through dynamic coupling of macroscale and CG models. We shortly described a back-mapping algorithm that converts a DTS topology to a Martini structure. This algorithm opens up a new perspective to perform a dual resolution Martini/DTS simulation, so that DTS performs the large-scale moves while local moves of the chemical components is handled by the CG Martini model.

## Data Availability

The datasets generated for this study are available on request to the corresponding author.

## Author Contributions

All authors listed have made a substantial, direct and intellectual contribution to the work, and approved it for publication.

### Conflict of Interest Statement

The authors declare that the research was conducted in the absence of any commercial or financial relationships that could be construed as a potential conflict of interest.
